# Addressing Barriers to the Development and Adoption of Rapid Diagnostic Tests in Global Health

**DOI:** 10.5772/61114

**Published:** 2015-01-01

**Authors:** Eric Miller, Hadley D. Sikes

**Affiliations:** 1 Department of Chemical Engineering, Massachusetts Institute of Technology, Cambridge, USA

**Keywords:** Rapid diagnostic tests, infectious disease, immunochromatography, point-of-care, global health

## Abstract

Immunochromatographic rapid diagnostic tests (RDTs) have demonstrated significant potential for use as point-of-care diagnostic tests in resource-limited settings. Most notably, RDTs for malaria have reached an unparalleled level of technological maturity and market penetration, and are now considered an important complement to standard microscopic methods of malaria diagnosis. However, the technical development of RDTs for other infectious diseases, and their uptake within the global health community as a core diagnostic modality, has been hindered by a number of extant challenges. These range from technical and biological issues, such as the need for better affinity agents and biomarkers of disease, to social, infrastructural, regulatory and economic barriers, which have all served to slow their adoption and diminish their impact. In order for the immunochromatographic RDT format to be successfully adapted to other disease targets, to see widespread distribution, and to improve clinical outcomes for patients on a global scale, these challenges must be identified and addressed, and the global health community must be engaged in championing the broader use of RDTs.

## 1. Introduction

In 2010, approximately 10 million deaths worldwide were attributed to bacterial, viral or parasitic infections, and the vast majority of this mortality occurred in developing countries. [1] In part, this disproportionately heavy burden is due to the higher rates of disease incidence observed in low-income settings, which are causally linked to local consequences of poverty, such as abundant disease vectors, poor nutrition, limited access to sanitation and health education, and environmental determinants like indoor air pollution. However, the high rates of morbidity and mortality inflicted by these diseases are due to more systemic failings — namely, underdeveloped health infrastructure and severely limited financial resources that render communities and individuals incapable of bridging this health care gap.

Surveys conducted by the World Health Organization (WHO) have found that 72% of the African populace and 42% of the Asian populace is served by minimal or no infrastructure. [2] Under these conditions, the supply of electricity and clean water is either unreliable or nonexistent, local medical supplies and laboratory equipment are limited or totally unavailable, and medical staff are minimally trained or altogether absent. In many of these settings, the trappings of a modern and effective health system — vaccines, chemical prophylactics, therapeutics, informed medical consultation, and accurate diagnostic tests — are largely inaccessible luxuries, available only in distant, centralized clinics.

In such resource-constrained situations, the mere act of seeking diagnosis for a nascent condition can prove prohibitively expensive. For instance, in low- and middle-income countries, the average medical expenses and income losses faced by tuberculosis (TB) patients over the course of treatment can amount to more than 50% of their average annual income, and half of this cost is incurred in the process of seeking diagnosis. [3] In the absence of local community clinics, patients are forced to travel to regional clinics to seek medical consultation, and up until the roll-out of GeneXpert nucleic acid amplification tests (NAATs) in 2010, the most sensitive means for diagnosing TB required 6–8 weeks of cell culture and a follow-up visit. [4] During this time, patient conditions can worsen, friends and family can be infected, and presumptive treatment can be initiated, increasing the risk of spreading drug resistance. [5]

For vast swathes of the global population, however, diagnostic consultation never even occurs. In 2012, for instance, the WHO estimates that 34% of all incident cases of tuberculosis — 2.9 million new infections — went undiagnosed, or were not reported to a central authority. [4] In the same year, approximately 36% of all incident cases of malaria — 74.5 million new infections — are thought to have gone undetected. [6] Diagnostic gaps such as these pose a severe threat to global health, inflicting heavy tolls of morbidity and mortality upon low-income communities, wasting valuable medical and financial resources on inappropriate medicines, and allowing infectious diseases to continue to proliferate unchecked.

Low-cost diagnostics made available at the point-of-care (POC) have been heralded as a means of closing these diagnostic gaps, by providing patients and local health care practitioners with timely, accurate and actionable diagnostic data that can inform clinical decisions and ultimately improve patient outcomes. These broadly distributed diagnostic resources also play a crucial role in preventing the further spread of drug resistance, and are critical for creating the accurate epidemiological models needed to efficiently direct resource distribution and elimination campaigns.

Rapid diagnostic tests (RDTs) are leading candidates to fill this point-of-care diagnostic niche. RDTs are diagnostic assays which can rapidly indicate the presence of pathogens or pathogen-associated biomarkers in patient samples. For the purpose of this review, we will focus on the most commonly employed form of RDT, immunochromatographic test strips. These tests, also referred to as “lateral flow assays,” use membrane-immobilized affinity agents to screen for the presence of antigens in peripheral patient fluids. The presence of the targeted antigen in a patient sample is signalled by the development of a coloured test line visible to the unaided eye within 5–30 minutes, and this result is taken to be indicative of infection. Immunochromatographic RDTs typically come in dipstick or cassette formats, and can be engineered in a multiplexed fashion, simultaneously testing for the presence of multiple antigens using a single device.

RDTs are well suited for use in resource-limited settings, in that they fulfil the ASSURED criteria, potentially demonstrating all of the characteristics considered essential for diagnostics intended for application at the point-of-care. To varying degrees, RDTs are: 1) **A**ffordable by those at risk of infection, 2) **S**ensitive (few false-negatives), 3) **S**pecific, (few false-positives), 4) **U**ser-friendly, (simple to perform and requiring minimal training), 5) **R**apid (to enable treatment at first visit) and **R**obust (not requiring refrigerated storage) 6) **E**quipment-free, and 7) **D**elivered to those who need it. [7, 8]

The development of RDTs is ongoing for many infectious diseases, ranging from HIV and syphilis, to influenza and tuberculosis, to visceral leishmaniasis and dengue. [9–14] Malaria RDTs, in particular, have seen significant technical development and market uptake. Within the field of malaria diagnostics, there are currently more than 200 commercially available products, [15] and in 2013, 319 million malaria RDTs were sold in 48 different countries. Of these, 160 million tests were subsidized and distributed within the public sector by national malaria control programmes. [16]

However, despite the promise that RDTs have shown for playing the role of the ideal POC diagnostic test, and despite the technological maturation seen in RDTs targeting malaria, their broader development and uptake by the medical community has been limited by a number of lingering issues. These range from technical and biological shortcomings – which have served to diminish device performance and undermine the credibility of RDTs with patients and health care workers – to social, infrastructural, regulatory and economic issues, which have slowed their distribution and adoption at the national and community levels.

This work will identify these obstacles, explore their mechanistic roots, and highlight current research and future actions that may serve to break down barriers to rapid diagnostic test development and adoption. Throughout this discussion, the rise of malaria RDTs will be taken as an illustrative case study, and the wealth of research into the subject that has thus far been conducted will be frequently referenced and used to inform recommendations for the advancement of RDTs as a more effective technological platform.

## 2. Technical Barriers

**Figure 1.**
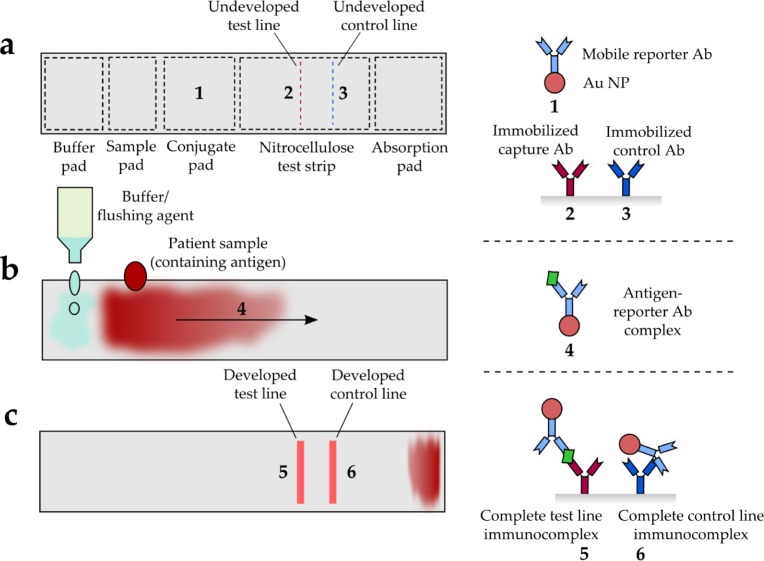
Standard configuration and mode of action for an immunochromatographic RDT. Note: Ab=antibody, Au NP=gold nanoparticle.

In a standard, single-antigen immunochromatographic RDT, the test strip is composed of several sub-elements (Figure 1a), and processing occurs in two steps. First, a predefined volume of the specimen (e.g. whole blood, plasma, serum, urine, sputum, etc.) is applied to a sample pad, followed by a washing step in which a buffer formulation is added either to a separate buffer pad upstream of the applied specimen, or directly on top of the sample. This fluid bolus migrates along the length of the test strip, drawn by capillary forces imposed by the downstream absorption pad, and first encounters the conjugate pad (Figure 1b). The conjugate pad contains antibodies that are coupled to reporter species such as microbeads, dye-cleaving enzymes or, most commonly, gold nanoparticles. The reporter antibodies in the conjugate pad are not surface-immobilized, and thus are free to travel with the fluid front. If present, antigens in the patient sample form complexes with these reporter antibodies. These complexes wick through the nitrocellulose membrane until they contact a thin band of immobilized capture antibodies, called the test line. These capture antibodies bind to a unique epitope of the targeted antigen, and the formation of a full immuno-complex (Figure 1c) leads to the concentration of reporter species, resulting in an optical readout visible to the unaided eye. Reporter antibodies that are not bound at the test line continue to wick along the membrane until they are bound by antibodies immobilized in a downstream control line (Figure 1c). It is important that the reporter antibody does not bind to the test line antibody unless the antigen is present to bridge the two antibodies, and that the reporter antibody does bind to the antibody used in the control line. The development of the control line confirms that the reporter species have mobilized and have maintained binding activity, and that the patient sample has been drawn across the test line.

Although seemingly simple in theory and design, RDTs are founded upon several core technologies that are subject to a number of potential technical vulnerabilities. [17] These technical complications can render tests either inoperable or at least unreliable, resulting in diminished test performance in the field. Common technical difficulties include insufficient component stability under thermal stress, inadequately characterized affinity reagents, the potential for erroneous readings due to subjective interpretation, the time-dependence of signal development, insensitivity to low disease loads, and unsuitability for pathogen quantitation.

### 2.1 Susceptibility to Heat and Humidity

One issue common to all RDTs targeting diseases endemic to tropical or sub-tropical regions is the potential for degradation due to heat and humidity. Extended exposure to these conditions can drastically reduce the shelf life of RDTs, causing warping or degradation of the nitrocellulose membrane, the hydrolysis of capture antibodies from the membrane or cleavage of reporter species from the reporter antibodies, and the irreversible denaturation of the capture or reporter antibodies. In order to preserve test integrity, RDT manufacturers recommend continuous product refrigeration, from the point of manufacture all the way to rural clinics and the point of use. However, in many developing countries, cold-chain continuity cannot be guaranteed, due to both a lack of infrastructure and variable observance of manufacturer recommendations along the supply chain.

Most manufacturers guarantee device stabilities within certain reasonable temperature ranges — recent WHO analysis of commercially available malaria RDTs found that 37 out of 50 tests claimed stability ranges of 2–30°C, and that the remaining 13 tests claimed stability up to 40°C. [18] However, in many countries, ambient temperatures regularly exceed these limits. Studies tracking a shipment of malarial rapid diagnostic tests to sites in Cambodia found that devices were exposed to temperatures in excess of 30°C for 28% of the time spent in transit and storage — roughly 3,400 hours. Included within this sum were 44 hours during which temperatures soared to between 40 and 45°C. [19] Even these conditions, however, are mild in comparison to the extremes typically seen in regions of sub-Saharan Africa. In a study conducted in Senegal, devices left at one site experienced temperatures in excess of 40°C for 18% of the time, and were stored at over 30°C for 89% of the time. Similar results were found in Ethiopia, where devices in one site were stored at temperatures over 30°C for 80% of the time. [18]

Extended exposure to these conditions has been linked to reduced test sensitivities. One site in Brazil found that after fifteen months' storage under ambient conditions, RDT test lines specific to the malarial species *Plasmodium falciparum* (*Pf*) and *Plasmodium vivax* (*Pv*) exhibited sensitivities of 79.7% and 85.7%, respectively, which is much lower than the 95% threshold recommended by the WHO. The authors attributed this reduced performance to extended storage in conditions outside of the recommended temperature range — the average maximum daily temperature at which the tests were stored was 29.4°C, with a range of 22.7 to 33°C. [20]

In order to minimize test attrition due to thermal degradation, successful RDT inventory management requires close coordination between manufacturers, governmental agencies, couriers and regional clinics, to provide seamless cold-chain continuity during the course of product distribution and storage. RDTs should be handled by the same standards established for vaccines and therapeutics, including the use of vaccine vial monitors for indicating the extent of thermal stress to which the products have been exposed. [21] Finally, if possible, lot performance should be tested regularly to detect potential product deterioration. [10]

Studies have been conducted into the use of evaporative cooling boxes (ECBs), which passively maintain reduced internal temperatures by harnessing the latent heat of evaporation. In Cambodian field sites, researchers observed that the use of ECBs resulted in an average 97% reduction in the number of hours *Pf*-specific RDTs were stored above manufacturer limits (30°C), versus storage under ambient conditions. As a result, the lifetime of the RDTs stored in ECBs was extended from 210 days to 300– 360 days, depending on the location. [22] Similar results were obtained in a study conducted in Afghanistan — *Pv*-specific RDTs stored in a passive cooling device were subject to temperatures that were, on average, 8°C cooler than ambient conditions, and as a result tests were never stored at temperatures greater than 30°C. This resulted in a test strip invalidity rate of only 10%, as compared with 17% for RDTs stored under ambient conditions. [23]

The climates in which RDTs are deployed are also subject to high humidity, which can impair device performance. [24] In a survey of 50 commercially available malaria RDTs and 11 HIV RDTs, it was found that almost all were hermetically sealed and shipped with desiccant sachets, in accordance with WHO guidelines. However, only 60% (30) of the malaria RDTs and 45% (5) of the HIV RDTs were observed to contain humidity-indicating dye, and of these, all 30 malaria RDTs and three of the HIV RDTs were found to contain the toxic dye cobalt dichloride, rather than the preferred, more benign, methyl violet or iron salts. Less than half of the products packed with self-indicating sachets instructed users to check the colour of the silica desiccant to assess test validity. [25] These changes — universal inclusion of benign, self-indicating desiccants and clear instructions for use — would serve to reduce the risk of RDT invalidation by humidity.

### 2.2 Inadequate Characterization of Affinity Agents and Antigens

An additional technical issue that has potentially contributed to the historically sub-optimal performance of commercial RDTs is the use of poorly characterized commercial antibodies that have not been optimized for use in low-infrastructure settings or with complex biological samples. The most frequently used affinity agents are monoclonal and polyclonal antibodies (both IgG and IgM), and these immunoglobulins are typically supplied by outside vendors removed from the RDT production process. Thus, there has generally been little oversight into commercial antibody development and the potential failure modes that can occur during this procedure.

The generation of antibodies for diagnostics is a complex undertaking. If a polyclonal mixture is being produced, the process involves the inoculation of a large number of animals with purified recombinant antigen and the subsequent harvesting of the antibody pool from the animals' blood or (in the case of chickens) egg yolks, via affinity chromatography. [26] One of the benefits of employing polyclonal antibodies is that this purified pool is likely to contain species exhibiting a distribution of biophysical characteristics, providing redundancy if individual clones should fail due to thermal denaturation. [27] This pool is also composed of clones that cumulatively recognize many antigenic epitopes, and this can result in strong, polyvalent binding interactions. [28]

However, the traditional production process for polyclonal antibodies is markedly imprecise, leading to the generation of clones specific to serum components and impurities within the original antigen preparation, as well as to conserved antigenic epitopes observed in closely related, environmental pathogens. This can result in reduced specificity if orthologues to the target antigen are ubiquitous, or if retained clones are specific to protein species found in patient samples. Although negative selections against known orthologues and host cell impurities can be conducted in additional affinity chromatography [28] or membrane purification [29] steps, the lack of clonal characterization and the absence of sequence information make direct manipulation of the polyclonal pool difficult. Additionally, the production process can be inconsistent — each new polyclonal pool is unique in its compositional make-up, and thus batch-to-batch variability in performance can be observed. [30]

In instances where antigen specificity and consistent antibody properties are concerns, monoclonal antibodies are employed in the place of polyclonal blends. Whereas the production of polyclonal antibodies takes only a few months, however, traditional monoclonal antibody development can take up to a year, and is considerably more costly. [31] Within this workflow, the lymphatic B cells of inoculated animals are harvested and analysed with single cell resolution, and the cell found to produce high-affinity antibodies specific to the antigen of choice is used in the creation of an immortalized hybridoma. This allows for the consistent, indefinite production of a well-defined clone exhibiting complementarity for a single antigenic epitope. However, while this reduces cross-talk with environmental protein species, it also introduces the risk that the identified clone lacks the robustness necessary to withstand the harsh conditions to which RDTs are exposed in the field. Thus, the heat lability of developed monoclonal antibodies should ideally be characterized via differential scanning calorimetry or thermal incubation followed by an immunoassay challenge. [32]

In addition, the reliance upon animal hosts for the production of antibodies raises concerns about whether the purified recombinant antigen used in immunization will present native, clinically relevant epitopes *in vivo*. The chemical and biological environment in which an antigen would naturally be detected in a patient's sample is potentially very distinct from the environment of an animal host's circulatory system. If these environmental differences elicit structural changes in the antigen species, the antibodies raised against it may not bind to the antigen in clinical samples with equivalent affinity. For instance, in the case of the malarial antigen histidine-rich protein 2 (HRP2), it has been shown that the secondary structural elements of the native, haem-bound form are drastically different from that of the unbound form. [33] Given the abundance of free haem in animal serum, this likely has little effect upon the suite of antibodies generated by the host immune system, but for antigens found in other peripheral fluids such as urine or sputum, structure-altering interactions with fluid-specific ligands or other chemical determinants may not be recapitulated in the host circulatory system.

The imprecision of polyclonal antibody production, the complexity of the monoclonal antibody identification process, and the uncertainty involved in using live animal immunization to generate antibodies provide the motivation for the use of protein display technologies for the generation of affinity agents. The methods most commonly employed are phage [34] and yeast [35] surface display, though *in vitro* techniques such as ribosome, mRNA, covalent DNA [36] and liposome [37] display have also been demonstrated. These methods are all founded upon the idea of screening vast combinatorial libraries for binding molecules, while maintaining a physical linkage between phenotype (i.e., binding properties) and genotype (i.e., the gene encoding a specific protein variant). Protein display technologies allow for the isolation of high-affinity binding molecules — immunoglobulins as well as non-antibody scaffold proteins — within the span of a month, enabling precise clonal characterization and straightforward genetic manipulation. Successful selections have been demonstrated against many targets, including biomarkers for infectious disease [38], membrane proteins [39] and whole cells [40]. Display technologies have also been used to engineer affinity agents that exhibit greater thermal stability. [41]

The use of these display technologies enables the generation of monoclonal or oligoclonal binding molecules within clinically relevant contexts. If made the industry standard, this practice would allow for greater precision and control in the development of commercial affinity agents for RDTs. Well-characterized oligoclonal pools of binding molecules are of particular interest, as they combine the specificity of monoclonal binders with the increased stability of a population of affinity agents. However, the mode of antibody production is only one-third of the problem — there are also technical issues to be addressed in the sourcing of target antigens, as well as in the selection of binding modalities.

Recombinant antigen sourcing is of utmost concern in the antibody production process. In many cases, commercial antigens are expressed as fusion proteins for soluble presentation [42], and the presence of this fusion partner (e.g., SUMO, NusA, MBP, GFP, etc.) can result in the generation of non-specific antibodies that can only be culled via negative selections against the purified, solitary fusion species. Protease cleavage techniques can be employed for fusion tag removal following soluble protein expression, but these processes tend to be inefficient. To date, the highest cleavage efficiencies have been demonstrated by the SUMO tag/protease system. [43] Another critical consideration in the preparation of antigens for antibody generation is the glycosylation state of the protein. If expressed in *E. coli*, eukaryotic protein species will lack their native glycosylation, and even eukaryotic expression systems such as *P. pastoris* could potentially yield non-natural glycosylation profiles. [44] Furthermore, heterologous protein expression systems have been shown to be subject to the formation of insoluble inclusion bodies [45] and the mis-incorporation of amino acids [46], both of which can result in misfolded products. Thus, the expression system used to produce recombinant antigens must be chosen with careful consideration of the antigen's native host, and protein characterization studies (e.g., circular dichroism, size exclusion chromatography, and mass spectrometry) should be conducted prior to binder selection.

Increasing focus has also been given to the potential use of non-immunoglobulin affinity agents in RDTs. Antibodies are bulky macromolecules, which limits their effective surface density upon immobilization and renders them susceptible to non-specific binding by heterophilic antibodies and other blood factors. [47, 48] Immunoglobulins have also been shown to be inherently unstable molecules, losing binding activity following surface immobilization even in ideal, refrigerated conditions. [49] Finally, they are also subject to a restrictive intellectual property landscape, which reduces the possibility of patent protection for innovative antibody-based diagnostics. [50]

In order to circumvent these limitations, a number of alternative affinity agents such as ankyrin repeat proteins, single-domain camelid antibodies, oligonucleotide aptamers and shark immunoglobulins have been investigated for use in diagnostic tests for HIV, [51] dengue, [52] tuberculosis, [53, 54] and malaria. [55, 56] Given the innate thermostability and bio-orthogonality of these novel binding scaffolds, it is expected that their use in commercial RDTs could yield significant stability and specificity benefits in comparison with antibodies. Accordingly, it is expected that interest in their potential uses in diagnostic applications will only continue to grow in coming years.

### 2.3 The Prozone Effect

An additional possible source of technical failure is the prozone effect — the occurrence of false-negatives or inaccurately low-intensity test lines as a result of 1) overabundant antigens or 2) overabundant antibodies. In the first case, unbound antigens may completely saturate the test line, preventing the binding of antigen-conjugated reporter complexes, and the development of a positive signal. In the second case, antigens can become masked in multivalent immune complexes, resulting in reduced antigen binding at the test line. [57] This phenomenon was found to be common within certain test formats — in a study conducted upon HRP2-specific RDTs, 16 out of 17 tests demonstrated the prozone effect, although the same study found that RDTs specific to parasite lactate dehydrogenase (pLDH, another malarial biomarker) yielded no incidence of the phenomenon. [58] The effect only occurs in samples exhibiting high parasitaemia, with more than 4% of red blood cells parasitized, and the incidence of the prozone effect within these high-load samples ranged from 6.7–38.2%. [59]

A number of strategies can be employed to prevent the occurrence of false-negatives due to the prozone effect. Firstly, manufacturer recommendations on the appropriate sample volume to be applied should always be followed, as application of too large a volume can elicit the phenomenon. [58] The prozone effect is also abated upon dilution of the patients' samples, and so for patients presenting severe clinical manifestations of malaria, health care workers can elect to verify a weak or negative test result using a diluted sample. [60] Finally, the use of alternative device architectures may prevent the occurrence of the prozone effect. The Dual Path Platform is a novel immunochromatographic format in which the patient sample and reporter antibodies are mobilized separately and sequentially, and travel along distinct paths en route to the test line. This allows for any antigens present in the patient sample to bind to the capture antibodies prior to conjugation with the reporter complexes, and thereby precludes the possibility of prozone-mediated false-negative results. [61]

### 2.4 Subjective Interpretation of Test Lines

By virtue of being targeted for use at the point-of-care, immunochromatographic RDTs must be amenable to facile and accurate interpretation by minimally trained operators. However, test lines are often subject to highly variable and user-dependent readings, which can be impacted by user biases, low signal contrast, and inadequate training materials. Additionally, tests can yield anomalous results that can be difficult to interpret accurately, frequently leading to erroneous diagnosis. [62] Over the course of the WHO analysis of commercial malaria RDTs, a broad variety of abnormalities were observed, ranging from irregular migration of the fluid front that obscured faint signals, to “ghost lines” in which test lines remained uncoloured, and the background became heavily stained. [63] In order to reduce the impact of these phenomena and counter the subjective nature of visual evaluation, developed tests should be read by multiple independent interpreters, and the level of agreement assessed. [64]

A number of additional strategies have also been proposed for reducing inter-user variability in test interpretation. Firstly, package inserts must include clear examples of all potential test outcomes, particularly in the case of multiplexed assays, and must address the appropriate course of action to take upon the development of faint test lines. [15] Universal training materials should also ideally provide case studies by which the accuracy of operators' interpretations can be tested. [65]

It has also been suggested that, given the ubiquitous presence of camera phones, cellular devices could be leveraged as a means of relaying test results to centralized laboratories, where they could be digitally processed and analysed by trained technicians. [66] This would not only serve to reduce local operator error in test interpretation and enhance test sensitivity, but could also provide valuable epidemiological information to regional clinics. However, a number of possible issues exist with this approach, including the digital transmission of sensitive personal medical data, the potential for incompatibility with the camera phone interface owing to the diverse and varied nature of RDTs, and possible test-to-test variability due to local lighting conditions and user practices. [61]

**Figure 2.**
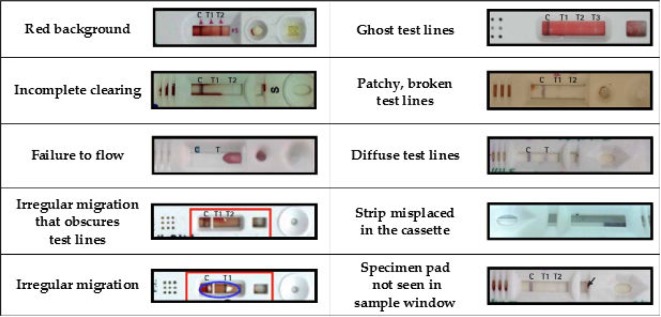
Malaria RDT anomalies encountered in production lots. Figure reproduced with permission from reference 63.

Interpretation of test results may also be facilitated by a reduction in the background signal, either by de-colouring patient samples, as has been demonstrated with whole blood, [67] or by preventing non-specific adsorption of patient sample components to the test membrane. Systematic studies of the effects of membrane materials and porosity upon non-specific adsorption and the development of background signal are much needed, as non-fouling RDT materials could make a considerable difference in device performance. [68] Finally, while the method has not yet been adapted to the RDT format, polymerization-based amplification shows promise for allowing the development of high-contrast test results. The entrainment of phenolphthalein in nascent polymer thin film yields an intense purple signal upon basic treatment, and this has been shown to create a stark contrast, with low background signal. [69]

### 2.5 Time-Dependence of Signal Development

Test performance can also be negatively impacted by the interpretation of tests after the recommended time period for incubation has elapsed, or the archiving of tests for future reference, both of which can lead to the development of false-positive results. This delayed test line development likely occurs due to the upstream migration of free reporter conjugates and cross-reacting blood elements from the absorption pad — termed “backflow” — and their non-specific adsorption to the test line. [70] Manufacturers generally include recommended timelines for test development in the product literature, but these guidelines may not be heeded if instructions are unclear, timing devices are in short supply, or regional clinics are understaffed and clinicians are otherwise occupied.

Several solutions to this problem have been proposed. In situations where camera phones are available, digital archiving of test results obviates the possibility of further post-test development. Modular RDT configurations have also been demonstrated, allowing for lateral flow test elements to be snapped into place, and for the physical isolation of the test line from the remaining sample bolus following signal development. [71] Additionally, the strategic inclusion of evaporation windows in the cassette housing surrounding the absorption pad can reduce the incidence or extent of back-flow. [72] Finally, alternative methods of signal amplification which do not rely upon the optical concentration of bulky nanoparticles may serve to reduce the potential for non-specific adsorption of colloidal reporter conjugates to the test line, and allow the further development of archived samples.

Polymerization-based amplification has been proposed as a means for the temporal discretization of test line development. Within this system, reporter antibodies are coupled to small molecule photoinitiators rather than to gold nanoparticles. Photoinitiators trigger a rapid chemical reaction upon exposure to visible light, yielding a polymeric thin film from aqueous monomer species within seconds, and the film is visible to the unaided eye. The discrete nature of this signal amplification process, controlled by a pulse of light, eliminates the potential for extended test line development, and the initiator-coupled reporter antibody may exhibit a reduced propensity for non-specific adhesion to the nitrocellulose membrane in comparison with antibodies coupled to bulky nanoparticles, which can become entrained in viscous samples such as sputum or saliva. [69, 73]

### 2.6 Insensitivity to Low Disease Loads

The technical challenges discussed in the preceding sections, in combination with a number of biological determinants, are implicated in the modest limit of detection demonstrated by many RDTs, which renders them insensitive to low pathogen/antigen loads. This shortcoming represents a significant challenge to the diagnostic format and reduces its potential utility in elimination programmes, in populations exhibiting inherently low pathogen loads, and in patients seeking diagnosis during critical, quiescent stages of the pathogen life cycle. [74] In the case of malarial RDTs, the WHO requires a panel detection score of 75% at a parasitaemia of 200 parasites/μl in order for the product to warrant endorsement. [16] However, the threshold for pyrogenic onset has been shown to vary from 10 to 200,000 parasites/μl, with 22% of patients developing their first acute fever at a parasite load below 200/μl. [75] Thus, even for the select assortment of commercial malaria RDTs demonstrating compliance with WHO standards, sufficiently low limits of detection have not been demonstrated for the early and accurate diagnosis of nascent infections, or of asymptomatic carriers.

Validating the need for lower limits of detection in RDTs, one study of healthy Gabonese adults with no febrile symptoms found that more than half of participants nonetheless tested positively for *P. falciparum* malaria via nested polymerase chain reaction (PCR). [76] These samples were then tested by RDT, using a product with an 80% panel detection score from the relevant round of WHO product testing, [77] and the RDT sensitivity using PCR as the reference standard was observed to be only 46%. In all cases of a false-negative result, the parasite load was observed to be 100/μl or less, demonstrating the inadequacy of RDTs when operating at these levels.

Several methods have been developed to improve the inherent limits of detection for diagnostics, many of which could find eventual application in commercial RDTs. One group has recently demonstrated the use of a simple pre-processing step to concentrate the malarial antigen HRP2 prior to RDT application, resulting in a four-fold signal increase and a limit of detection as low as three parasites/μl. [78] A similar process has been used to concentrate tuberculosis bacilli in sputum using magnetic nanoparticles. It has been proposed that this method, used in conjunction with a lysis protocol, could be used to locally concentrate intracellular biomarkers. [79, 53] This approach may also prove fruitful in the diagnosis of enteric fever and human African trypanosomiasis, as both diseases require pathogen concentration for sensitive detection. [80]

Alternative device architectures have also been considered for enhancing the limits of detection for RDTs. Immunochromatographic formats incorporating multiple input streams are claimed to increase test sensitivity by allowing patient antigens to form stable binding interactions with the capture antibodies prior to being bound by reporter complexes. This reduces the incidence of multiple antigens binding to single reporter antibodies, and allows for test line saturation. Examples of these novel formats include the previously mentioned Dual Path Platform and three-dimensional stacking flow architectures. [61, 73] Finally, the functionalization of glass slides with non-fouling “polymer brushes” has been shown to greatly reduce the level of non-specific adsorption in immunoassays, yielding femtomolar limits of detection in complex media. [68] If adapted for use in an immunochromatographic format, this technique could potentially render RDTs capable of playing a key role in elimination campaigns and early case detection.

Additionally, the identity of the bio-conjugates employed in immunochromatographic assays impacts the inherent test sensitivity — in a comparison between four different reporter conjugates, it was found that carbon black nanoparticles exhibited the lowest limit of detection by an order of magnitude. [81] RDTs based upon cellulose nanobeads have also been shown to yield higher sensitivity than those based upon colloidal gold reporters, due to the increased surface-area-to-volume ratio of the cellulose particles, and the greater degree of optical contrast afforded by the incorporation of dye molecules into the nanobeads' porous structure. [82] The use of immunoliposomes has also been shown to drastically improve the limit of detection, due to the liposomes' ability to internalize high concentrations of absorbent or fluorescent dyes. [83] Other labelling candidates that may improve test sensitivity include silica nanoparticles, quantum dots, silver-enhanced colloidal gold, magnetic nanoparticles, dendrimers and carbon-based structures such as nanotubes and colloidal carbon black. [84]

Finally, efforts to optimize the paratopic stability and orientation of surface-bound affinity agents may yield lower limits of detection, by increasing the proportion of active capture agents participating in the biorecognition event. One approach to improving surface-bound reagent stability involves the addition of a disaccharide such as sucrose or trehalose to the protein formulation during lyophilization. This results in the formation of an amorphous sugar matrix which places conformational constraints upon the embedded proteins, thereby preserving the integrity of critical paratopes. [85] Recent studies have also demonstrated the use of multivalent display platforms — such as lyophilized surface-displaying yeast cells and self-assembled protein particles — for the stable, preferentially oriented presentation of capture agents. [86, 87] These approaches may enhance the intrinsic activity of immobilized test line reagents, rendering RDTs more sensitive to low disease loads.

### 2.7 Unsuitability for Pathogen Quantitation

A further technical issue facing RDT manufacturers is the growing desire within the medical community for quantitative or semi-quantitative diagnostic results. The ideal diagnostic test would allow accurate quantification of a patient's pathogen load, as this information would enable clinicians to make more informed treatment decisions based on knowledge of the probable stage of disease progression or its measured response to therapeutic treatment. Thus far, commercial RDTs have been strictly binary, the antigen being either absent from a patient sample or present to an arbitrary degree. Studies have shown, however, that test line intensity can be loosely correlated with antigenaemia, [16] particularly if fluorescent reporter species such as dye-loaded liposomes are used. [88] At the expense of introducing equipment and technical infrastructure, the use of digital image processing could allow for semi-quantitative analysis of these RDT results in centralized medical centres. [89] Additionally, titrations of increasing antibody concentrations immobilized on membrane surfaces in a bar code pattern might be used as a semi-quantitative measure for bracketing antigen levels. [90]

Ultimately, however, RDTs are antigen tests, and thus in order for any quantitative or semi-quantitative measure of RDT signal output to be of clinical utility, the concentration of the antigen in the patient sample must correlate with the actual pathogen load. A number of studies of the malarial antigen HRP2 have attempted to model this relationship both *in vitro* and *in silico*, and have claimed that its concentration in patient samples can be approximately correlated to malarial parasitaemia, even in cases where the bulk of the pathogen load is sequestered within the patient capillary system. [91, 92] However, it has been shown in patient samples that the same level of parasitaemia can result in drastically different plasma concentrations of HRP2. [93] This might be due to differences in the life cycle stage between different samples, holdover of HRP2 from previous infection, immunocomplexing of the antigen, or even just stochastic effects in the productivity rates of different parasite populations.

Molecular diagnostics such as NAATs and quantitative PCR may allow for the straightforward quantitation of actual disease load due to the direct proportionality between gene representation and pathogen levels. It must be noted, however, that even molecular quantitation is complicated by the possibility of pathogen sequestration or latency, in which case pathogen loads would be underestimated. [76] Ultimately, protein expression levels have proven difficult to reliably relate to pathogen loads, and thus even if RDTs are made suitable for the approximate quantitation of antigen levels in patient samples, these results may not allow the accurate quantitation of the associated disease load for reasons relating to the underlying biology of the infectious agent and the host response.

## 3. Biological Barriers

These technical complications must be overcome in order for immunochromatographic RDTs to consistently demonstrate a high degree of test sensitivity. However, the mechanics of test operation are only one part of the problem — the performance of an RDT is inherently limited by the quality of the disease biomarkers that it is designed to detect, as well as by the nature of the sample with which it is being challenged. These biological issues contribute to poor device performance, and represent barriers to the realization of sensitive and specific rapid diagnostic tests.

Box 1 Characteristics of the ideal biomarker of infectious disease – SASQUATCH

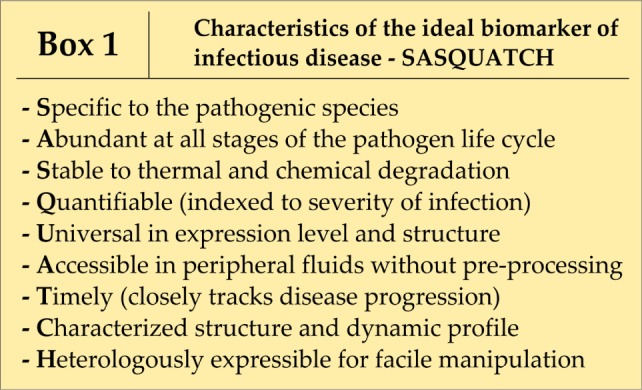

– **S**pecific to the pathogenic species
– **A**bundant at all stages of the pathogen life cycle
– **S**table to thermal and chemical degradation
– **Q**uantifiable (indexed to severity of infection)
– **U**niversal in expression level and structure
– **A**ccessible in peripheral fluids without pre-processing
– **T**imely (closely tracks disease progression)
– **C**haracterized structure and dynamic profile
– **H**eterologously expressible for facile manipulation

### 3.1 Non-Ideal Biomarkers

Much as the ideal rapid diagnostic test would fulfil ASSURED criteria, the ideal infectious disease biomarker to be targeted by an RDT would also meet a distinct and exacting set of benchmarks. We propose that the ideal biomarker must be:

- Specific (to the pathogen as well as to the particular species or serotype)
- Abundant (to a degree sufficient for detection, at all pathogenic loads, and during all stages of pathogen life cycle)
- Stable (thermally and chemically, and reliable)
- Quantitatively meaningful (indexed to pathogenic load to allow estimation of disease severity and tracking of therapeutic effect)
- Universal (in both expression and structure, geographically and amongst patients of all ages, genders, ethnic backgrounds and immune states)
- Accessible (in peripheral fluids of all patients, regardless of age, immune status or prior infection, and without sample pre-processing or concentration)
- Timely (observed in early stages of infection and eliminated quickly upon pathogen clearance)
- Characterized (well-understood physiological role, structure and dynamic profile)
- Heterologously expressible (in context-relevant form for facile generation of binding molecules and for use in assay development as a positive control)

By examination of the suite of biomarkers currently targeted by RDTs, it becomes evident that biomarkers fulfilling all of these SASQUATCH criteria are rare. In the case of malaria, HRP2 is absent within sub-populations in Peru and Brazil, and has highly heterogeneous primary sequence (not universal). [94–96] HRP2 also persists in the bloodstream for up to a month after parasite clearance (not timely), [97] and its accurate heterologous expression is difficult due to the potential for absent glycosylation motifs and amino acid substitution. [98, 46] HRP2 is, however, present in abundance, accessible in the patients' bloodstream, thermally stable, and highly specific to *P. falciparum*, and in this way it meets some benchmarks of ideality. [97]

In another example, lipoarabinomannan (LAM) is a lipopolysaccharide associated with the cell wall of *Mycobacterium tuberculosis* (*Mtb*). It is heat stable, found in urine samples and specific to active, but not latent, tuberculosis. However, LAM is not a protein species, which prevents its facile heterologous expression, and it features a conserved mannan core that is also observed in similar cell wall derivatives from environmental, non-pathogenic species of *Mycobacterium*. This is thought to result in cross-reactivity in RDTs, negatively impacting the biomarkers' specificity for *Mtb*. Furthermore, the sensitivity of LAM-based tests has proven to be poor, because the concentration of LAM in patient urine is strongly dependent upon the patient's immune status. In non-immunocompromised patients, LAM is bound within large immune complexes, preventing its passage through the renal glomerular basement membrane into the patient's urine. It is only in severely immunosuppressed patients that LAM is detected with any sensitivity, and thus the biomarker is considered to be of diagnostic utility only for HIV-positive individuals. However, even at CD4 counts of ≤50 cells/μl, reported test sensitivities are only 66.7%. Thus, a LAM-based test would at best serve only as an opt-in test for determining which patients have a high likelihood of harbouring tuberculosis. [99, 100]

In a third and final example, NS1 is a well-characterized biomarker of the dengue virus. It is a structural protein amenable to heterologous expression, it is present in abundance in patient serum during the first nine days after the onset of symptoms, and it is 100% specific to dengue infections. However, NS1 is of greater diagnostic utility for primary dengue infections than for secondary infections, as it becomes complexed by circulating host antibodies during secondary infections. This phenomenon decreases the sensitivity range of dengue RDTs from 94.7–98.3% for primary infections to a range of 67.1–77.3% for secondary infections. [101] This decrease in sensitivity represents a considerable health challenge, as secondary dengue infections can manifest as much more severe illnesses requiring more urgent and concerted care, such as dengue haemorrhagic fever and dengue shock syndrome. It is thought that this same phenomenon occurs in the case of the malarial antigen HRP2, with the host humoural immune response causing the sequestration and degradation of the HRP2 antigen. [102]

In order to improve test sensitivities when dealing with non-ideal biomarkers, manufacturers of RDTs often seek to use the multiplexed detection of distinct, species-specific antigens. In the case of malaria, RDTs are often multiplexed to detect HRP2 and the *P. vivax* variant of parasite lactate dehydrogenase (*Pv*-pLDH), to distinguish between infections by *P. falciparum* and *P. vivax*. Alternatively, tests can be multiplexed to select for *Pf*-pLDH and HRP2, which yields highly sensitive indications for *falciparum* malaria. [16] Multiplexed RDTs have also proven to be efficacious in discriminating between distinct diseases with non-specific symptomatic presentations. One recent study demonstrated the use of multicoloured nanoparticles to distinguish between three febrile illnesses — yellow fever, Ebola and dengue fever — with minimal non-specific signal development. [103] Finally, in dengue diagnosis, test sensitivity has been shown to be greatly improved by the multiplexed detection of NS1 with anti-NS1 IgG and IgM host antibodies, with sensitivity increasing from 69.2% to 93.0%. [104]

**Figure 3.**
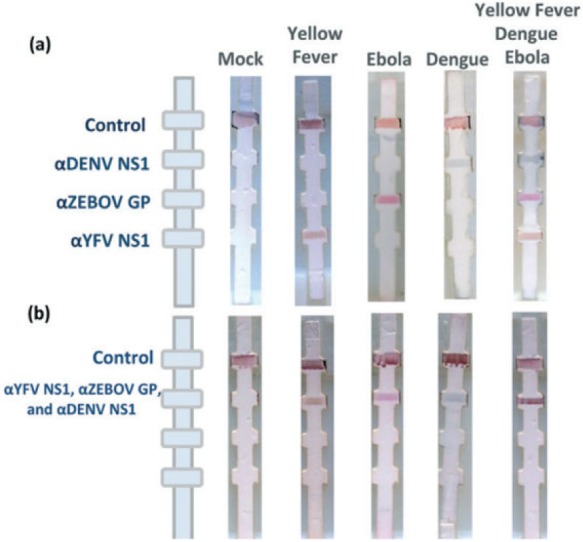
Multiplexed detection using silver nanoparticles (AgNPs). a) Antibodies to viral proteins are printed on the individual detection areas. A mixture of AgNPs conjugated to anti-dengue NS1, anti-Ebola GP, and anti-yellow fever NS1 antibodies is deposited in the conjugate pad. When each of the infectious disease biomarkers is detected, the corresponding AgNPs form a coloured band at the specific line. b) Capture antibodies are combined and printed into a single test line, which produces a band of a different colour depending on the antigen detected within the sample. YFV: ornage; EV: red; DENV: green. Figure reproduced with permission from Reference 103.

For many diseases, however, such antibody tests — that is, the use of a pathogenic protein in a test line to detect anti-pathogen host antibodies — are the only option currently available for RDT-based diagnosis, due to the lack of abundant, disease-specific biomarkers. This serological diagnosis is the standard for a broad diversity of diseases, including but not limited to HIV, chikungunya, leptospirosis, brucellosis and enteric fever. However, serological diagnosis is subject to a number of problems. Firstly, it is only an indirect means of diagnosing infection, as it uses the host immune response as a proxy for direct pathogen detection. As a result, serological tests yield a less reliable sense for pathogen burden, and only yield highly sensitive results if the targeted host antibodies are specific to a particularly immunogenic pathogen biomarker. Additionally, the time course of development for the host immune response can be variable — IgM antibodies for chikungunya infections only emerge one week into the course of illness, for instance, rendering RDTs useless until disease symptoms are fairly advanced. [80]

Serological tests can also exhibit low specificity. For instance, in the diagnosis of leptospirosis, the protein used to target anti-pathogenic IgM antibodies is a whole cell antigen prepared from a non-pathogenic strain of leptospire. Given the ubiquitous nature of non-pathogenic leptospires encountered environmentally (e.g., while farming), many patients may have already developed IgM antibodies to this antigen despite not harbouring a pathogenic infection, and this can result in cross-reactivity. [80] Additionally, the persistence of antibodies in host circulation (particularly IgG antibodies) can result in false-positive results after primary infection, which renders serological tests detecting IgG antibodies useless. False-negative results are also common when serological tests are used for the diagnosis of immunocompromised patients. [28]

In order for the next generation of rapid diagnostic tests to see the sort of proliferation and widespread use observed in malaria RDTs, the identification of a new suite of disease biomarkers is essential. Efforts to identify abundant, disease-specific protein species accessible in the peripheral fluids of patients are underway for many infectious diseases, using sensitive “omics”-based methods to detect pathogen-specific signals in noisy biological systems. [105] For instance, multiple groups have recently used a high performance liquid chromatography-mass spectrometry workflow to identify tuberculosis-specific protein antigens in patients' urine. [106, 107] If these antigens can be used in a multiplexed fashion for the sensitive and specific detection of active TB, it would allow for confirmatory RDT analysis of individuals incapable of producing sputum, such as young children and immunocompromised patients. It would also allow testing to occur without the production of potentially infectious bio-aerosols, which are hazards typically associated with sputum-based tests. [99]

### 3.2 Lack of Biomarkers/Drug Resistance

In a novel approach, several groups have also explored the potential of using probe molecules — “synthetic biomarkers” — as a means of interrogating the patient's disease state when ideal endogenous biomarkers are absent. One study demonstrated the use of thrombin-sensitive nanoparticles, which release ligand-encoded reporter molecules upon cleavage by the up-regulated proteases passively encountered in tumour tissues. These free reporter complexes are concentrated in the patient's urine, and can subsequently be detected by a paper-based immunochromatographic test. [108] An analogous, albeit less invasive, approach demonstrated the use of rational structural design principles to construct a synthetic fluorogenic probe that becomes activated upon cleavage by the *Mtb*-specific enzyme, BlaC. While this cleavage product was not reported in the context of an RDT, the study makes similar use of exogenous biomarkers in lieu of host or pathogen biomarkers. [109]

This synthetic biomarker approach may prove useful not only for indicating the presence of pathogens for which ideal native biomarkers have not yet been identified, but also for probing the drug resistance of these pathogens. If this drug resistance is affected by the activity of an enzyme that degrades a front-line antibiotic (such as a cephalosporin or β-lactamase), this enzymatic activity can be reported by way of a cleavable probe, which, as in Reference 108, is only accessible in peripheral patient fluids in its free state. The use of small molecule probes as reporters of antibiotic resistance have been demonstrated, [110] and if this approach for detecting drug resistance proves successful, it could address one of the key arguments levelled against RDTs by proponents of molecular diagnosis.

### 3.3 Cross-Reactivity in Complex Fluids

Another biological factor potentially contributing to the variable performance of rapid diagnostic tests is the potential for non-specific cross-reactivity between the test antibodies and elements found within complex biological samples. In validation of this idea, the WHO's fifth round of malarial RDT evaluation included a study in which products were challenged with blood samples that had been spiked with potentially cross-reacting compounds that might be observed in patient samples: anti-rheumatoid factors, nuclear antibodies, anti-mouse antibodies and rapid plasma reagin. While eight of the 42 products tested showed no cross-reactivity whatsoever, the remaining 34 products showed cross-reactivity to at least one of the compounds, to variable degrees. Anti-mouse antibodies posed the greatest challenge, leading to the development of false-positive results in all 34 cross-reacting products, followed by rheumatoid factors (29), anti-nuclear antibodies (26) and rapid plasma regain (15). [16]

A number of strategies have been proposed to reduce the incidence of these off-target reactions. As previously mentioned, negative selections against whole blood or cross-reacting compounds can be conducted to remove promiscuously binding antibodies from a polyclonal pool. [29] Additionally, buffer formulations can be specifically designed for the inactivation of certain contaminating elements of the patient sample. [111] Finally, the use of the aforementioned bio-orthogonal affinity agents may drastically reduce this cross-reactivity, by presenting reduced molecular footprints and protein epitopes to which host antibodies would not display complementarity.

## 4. Social Barriers

In the early stages of RDT development, the absence of harmonized regulatory oversight led to the proliferation of so-called “backyard” manufacturers, [14] and as a result, RDT markets became diluted with inexpensive, sub-optimal products. The prevalence of these low-performing devices has eroded the credibility of RDTs among health care workers and within the broader communities they serve, and has also complicated efforts to connect these communities with validated, higher-quality RDTs that come at a higher cost. As a result, there exist a number of social and economic barriers to the successful, widespread integration of high-performing RDTs into the diagnostic landscape.

### 4.1 Non-Compliance with Negative RDT Results

As with any new health technology, manufacturers and global health practitioners must work to educate the user base about RDTs, and must endeavour to correct any misconceptions that might have emerged over the past decade of technological development. In a study conducted in an area of Kenya where malaria RDTs were just being introduced as part of a pilot programme, the authors found that one of the primary challenges facing RDT introduction efforts was patients' poor adherence to negative RDT results. In regions where febrile illnesses have historically been presumptively treated as malaria, a negative RDT result can inspire incredulity, especially if community regard for modern medicine is unfavourable, or if patients are unfamiliar with, or distrustful of, the new test format. Consequently, patients receiving negative results by RDT were often seen to self-medicate despite those results, purchasing single-drug anti-malarials over the counter at local pharmacies, or using medicine leftover from previous infections. [112]

This suspicion of negative test results is shared by local health care workers, as well — a study in Uganda found that while 92% of clinicians believed that a positive malaria RDT result was sufficient confirmation of a malarial infection, only 49% believed that a negative RDT result was sufficient grounds to rule out the disease. [113] This reluctance to withhold anti-malarial treatment on the basis of an RDT result is particularly pervasive when dealing with children. In Zambia, 68.6% of febrile children with negative malaria RDT results were nevertheless prescribed the expensive artemisinin combination therapy (ACT) Coartem®. [114] Cohort studies have validated the safety of withholding anti-malarials from children, based on the high negative predictive value of RDTs. [115] However, health care workers' preconceptions and the perceived risk of a false-negative outcome still leads to habitual over-prescription of medications.

### 4.2 Non-Adoption due to Cultural Determinants

In many cases, social and cultural factors deterred individuals from submitting to RDTs at all. In a study conducted in two rural communities in Cote D'Ivoire, only 34 out of 100 self-presenting patients agreed to an RDT, and post-evaluation interviews found that this aversion was attributable to a variety of cultural determinants. Some patients were reluctant to submit to RDT analysis for fear that they were secretly being tested for more stigmatized illnesses, such as HIV. [116] This mentality was particularly prevalent in communities where malaria and HIV RDTs were concurrently available. Other patients declined RDT analysis on religious grounds, citing the sacred nature of blood, and a preference for traditional diagnosis and treatment, which are thought to address both the physical and spiritual aspects of the disease without requiring blood to be drawn.

In order to address these negative perceptions, it is essential that locally and culturally relevant programmes of information distribution, education, and counselling be enacted. The value of such programmes can be seen in the starkly contrasting response of well-informed communities in Ghana, where 98% of caregivers expressed a preference for malaria RDT-based case management of their child's febrile illness over presumptive treatment, even acknowledging that a negative result would mean that ACTs would not be prescribed. This high level of acceptance within the community was predicated upon an understanding that RDT use provides health workers with critical information that fundamentally guides and improves their treatment strategy. [117]

## 5. Infrastructural Barriers

The lack of general health education is a symptom of broader societal problems, namely a dearth of qualified health care workers and an underdeveloped medical infrastructure. These insufficiencies can lead to product misuse and supply interruptions, both of which serve to degrade product credibility and reduce its potential for impact in local communities.

### 5.1 Inadequate Training and Instructions

Though the device format is designed for use by individuals with minimal formal training, general literacy and an understanding of basic scientific principles are required for appropriate test administration. If RDTs are used in self-testing scenarios by patients lacking these skills, or administered by primary health workers unfamiliar with proper RDT usage practices, tests may be misused, leading to misdiagnosis or outright test failure, and the erosion of consumer confidence. [62, 111, 118] However, for health care practitioners demonstrating basic medical skills and provided with formal training, successful implementation of RDTs does not appear to be dependent upon the technician's inherent skill level. In Bangladesh, highly-trained laboratory technicians and less experienced field technicians demonstrated roughly equivalent performance in using dipsticks to test for cholera, [119] and local community health workers in Uganda were fully capable of satisfactorily administering RDTs and making diagnostic judgments for malaria and pneumonia following an eight-day training session. [120]

In order to facilitate the successful adoption of RDTs by health workers, instructions and package labelling must be clear, consistent and available in a range of relevant national languages, especially English, French, Spanish and Portuguese. [15] As much as possible, device design and usage should be harmonized between different manufacturers, to provide for greater ease-of-use and to reduce the potential for confusion or improper application. Where possible, instructions should be supplemented with clear pictorial representations, and general guidelines for proper RDT implementation should be translated and made widely available by international coordinating bodies such as the WHO. [62]

### 5.2 Supply Chain Disruption

Another infrastructural barrier impeding the widespread adoption of RDTs in global health practices is the frequent occurrence of supply chain disturbances in underdeveloped medical systems, and the significant impact these perturbations can have upon the availability of highvolume consumables such as RDTs. Health care workers cite this supply disruption as a deterrent for establishing RDTs as a cornerstone of their diagnostic programmes. [112] Cross-sectional studies conducted at 15 health centres across Mozambique illustrate the scope of the problem, documenting significant lost malaria RDT consumption — as a percentage of the monthly usage rates — due to product stock-outs. These lost consumption rates ranged from 0–149%, with the weighted average being 78% (i.e., the number of patients who were not served with an RDT due to stock-out was, on average, equal to 78% of the monthly number of patients who were served). [121]

These sporadic supply interruptions were attributed to several key drivers: 1) unrealistically optimistic expectations of the lead times required to receive new shipments at regional levels, 2) a failure to sufficiently prepare for seasonal increases in malaria RDT consumption and the increased difficulty of navigating flooded roads to deliver shipments, and 3) inadequate local compliance with local inventory management practices. These elements were all considered symptomatic of a broader, systemic problem — poor leadership at the national level and a resultant lack of standardization and timely communication between different levels of the supply chain. In order to decrease gaps in coverage due to supply fluctuations, international agencies such as the WHO must model and facilitate good practices in high-level resource allocation, and in coordination with national agencies and commercial suppliers.

## 6. Regulatory Barriers

The establishment of universal regulation and a standard process for RDT evaluation is critical for preventing markets from being subject to the sort of uncontrolled and unquantified product heterogeneity that has slowed the uptake of malaria RDTs over the course of the last decade. While indicators of manufacturing compliance such as the CE-IVD mark are important, they are more administrative measures than measures of product quality. Rather, procurement decisions should be based upon the endorsement of the WHO, which is founded upon active assessment of test performance. By playing an active and visible role in endorsing products for countries seeking RDT supplier contracts, the WHO can help to incentivize manufacturers to submit their products for evaluation and to price high-quality products competitively for the market.

### 6.1 Product Evaluation

A model for this successful market intervention exists in the WHO's exhaustive assessment of available malaria RDTs. From 2008 to the present, the WHO completed five rounds of malaria RDT evaluations, and the results suggested a broad spectrum of device performance. In testing rounds 2–5, only 65.6% (86/131) of products featuring *P. falciparum*-specific test lines performed within WHO specifications, with 34 products demonstrating panel detection scores (PDS) below the 75% threshold at 200 parasites/μl, and a further 11 tests demonstrating unacceptably high false-positive rates, greater than 10%. Similarly, only 42% (42/100) of *P. vivax*-specific test lines met WHO standards, with 56 products failing on the grounds of low panel detection scores, and an additional two products demonstrating false-positive rates greater than 10%. In combination products that claimed to test for both species, only 31 out of 77 products met recommended procurement criteria for both species; 32 products only achieved a satisfactory PDS for one of the two species, and 14 products failed to meet specifications for either species. In total, only 44.3% (59/133) of products met WHO specifications for all relevant test lines: 25 *Pf*-only products, and 34 combination *Pf-Pv* products. [63]

In collaboration with the Foundation for Innovative New Diagnostics (FIND), the WHO has established a searchable database [122] that allows users to screen these malaria RDTs by setting acceptable thresholds for performance metrics (PDS, maximum false positivity rate, maximum invalid test rate, etc.), and directly comparing the performance of tests that meet those criteria. Tools such as these are critical for informing the choices of national and regional control programmes, and in the future, these objective presentations of RDT performance should ideally be made widely available for all emerging disease targets. With expanded availability of reliable information and context-appropriate recommendations, [17] governments should be able to requisition RDTs based on programmatic needs, rather than being limited by cost or historical practices. [78]

As tests for new disease targets such as dengue, enteric fever and visceral leishmaniasis begin to achieve significant market penetration in coming years, the WHO's experience in successfully conducting assessments of malaria RDTs must be leveraged. These new tests will come from a variety of manufacturers, and will necessarily demonstrate a broad range of performance characteristics. For instance, in 2013, seven dengue RDTs were available on the market, and reports of their sensitivity and specificity already varied wildly, with ranges of 48.5–98.9% and 73.8–100%, respectively. [80] In order for these new RDTs to gain the confidence of the global health community, and in order for the scarce financial resources of national health agencies to be put to good use, emergent RDTs require the same sort of rigorous evaluation and validation afforded to malaria RDTs. [123]

### 6.2 Establishing Testing Standards

The shrinking operational budgets of the WHO diminish the possibility of running evaluation campaigns similar in scale to the malaria RDT trials for each new disease target. Instead, the WHO must focus upon standardizing studies conducted in academic, commercial and governmental labs, releasing guiding documents for study design [124] and exercising oversight on the conduct of these studies prior to accepting third-party results into composite data sets. Critical study design elements that warrant confirmation include the use of appropriate study groups in high-prevalence settings, comparison against an appropriate reference standard test and the conduct of appropriately blinded evaluations with adequate sample sizes. [123]

The establishment of universal reference standards are a point of particular concern, as many “gold standard” assays also suffer from sub-optimal sensitivities and specificities, and may not be practical in rural, endemic regions. For instance, the gold standard assay for the diagnosis of visceral leishmaniasis is the microscopic examination of splenic aspirates. While this technique yields adequately high sensitivities ranging from 93.1– 98.7%, the procedure is complex and highly invasive, requiring well-equipped medical facilities, trained and dedicated medical workers, and cooperative patients without demonstrable health complications. [13] In a case such as this, it would be infeasible for studies evaluating the performance of RDTs in endemic settings to use this diagnostic test as a basis for comparison, despite its being the most sensitive option available.

Thus, in order for the WHO to establish common standards across all evaluative studies for a given disease, consideration must be given not only to the performance of that diagnostic format, but also to its practicality and accessibility. If standards are to be established which are logistically prohibitive for rural settings, this must either be done at the conscious expense of RDT evaluation in those endemic regions, or the infrastructural and collaborative groundwork must be laid to enable local study facilitators to comply with these standards.

### 6.3 Quality Assurance

An additional challenge to the RDT platform that merits regulatory action is the need for mandatory quality assurance programmes and on-site lot evaluations. Significant lot-to-lot variation in malaria RDT performance has been observed, [1] possibly due to changes in manufacturing practices, membrane sourcing, or the batch of polyclonal antibodies used. To address this issue, the WHO and FIND currently offer free testing of malaria RDTs, and all parties — including manufacturers, centralized national distribution centres and rural clinics — are encouraged to submit samples for analysis. These RDT lot samples are maintained under conditions similar to those potentially encountered in the field and tested repeatedly throughout the lifetime of the product, in order to track any deterioration in product performance. [125] Although many suppliers do participate in the programme as part of a policy of continual process monitoring and improvement, use of these services is not mandated for WHO-endorsed manufacturers. Therefore, it is unclear how widespread this practice is, as well as whether participation in lot testing is broadly publicized.

As the RDT landscape develops for new disease targets, the continued expansion of these distributed RDT lot-testing capabilities is essential. It would also be of benefit to public health programmes to make WHO product endorsement contingent upon the manufacturer's commitment to continual lot-testing programmes. Additionally, further investigation into the failure modes that lead to inter-lot variability and performance deterioration is needed, and manufacturers should ideally be incentivized to investigate lot anomalies.

### 6.4 Positive Control Wells

Finally, it is unclear how frequently quality assurance services are taken advantage of at the community level, due to the logistical and financial challenges associated with shipping RDT samples to WHO testing centres and receiving results back. An approach closer to the ideal would be to provide for on-site testing of RDTs using standardized positive control wells (PCWs) containing lyophilized antigens. [126] PCWs for malaria RDTs have been developed by FIND, but the products are still under-going validation, one of the key concerns with their implementation being the potential for thermal degradation in rural health care settings. An appropriate strategy for addressing this issue might be to store positive controls in centralized, temperature-controlled facilities and to supply them intermittently to peripheral health clinics during regular on-site supervisory visits. [125] If they are made widely available by central regulatory agencies, the use of positive control wells would likely serve to increase confidence in test performance, and could play a key role in reducing over-prescription of medication.

## 7. Economic Barriers

Rapid diagnostic tests have been advanced as the ideal point-of-care diagnostic format, both for their simple and rapid operation, and for their affordability relative to alternative methods of diagnosis. However, systematic studies of their true economic benefit — both at the patientlevel and on the national scale — have thus far been limited almost strictly to analysis of the roll-out of malaria RDTs, starting in the early 2000s. Appropriate measures of the cost-effectiveness of these malaria RDTs must take into account not only their unit cost, but also more nuanced values such as cost savings achieved from reduced over-prescription of medications, reduced losses in patient income due to more localized diagnostic options, and the associated benefit to the broader national economy.

### 7.1 Cost-Effectiveness and Pricing

One study in Uganda found that at present, unsubsidized unit costs for malaria RDTs tend to fall within the range of US$2.54–2.83, which is much higher than the average value of $0.53 that consumers are willing to pay. [127] In order to ensure that RDTs are actually utilized at the community level, this price gap must be covered by external subsidies, most likely from the federal government. Studies show that at an 85% subsidy level, the rate at which RDTs are used to test febrile illnesses is doubled. [128] However, compliance with negative RDT results is only partial, with 49% of study participants over the age of five purchasing anti-malarial medications regardless of the test's outcome. This over-medication in response to distrust of negative RDT results drastically diminishes the cost-effectiveness of RDTs and results in 10–250% higher incurred costs for RDTs than for microscopic diagnosis, depending upon the local transmission rates. [129] While this measure of cost-effectiveness fails to account for the improved market penetration and accessibility of the RDT format, it reinforces the point that consistent compliance with test results is critical if RDTs are to yield their maximal financial and health benefits.

While RDTs have been shown to be of financial benefit in high disease transmission scenarios, in situations of lower prevalence their cost-effectiveness is less clear-cut. Programmes of mass screening and treatment (MSAT) based on malaria RDTs have limited efficacy in reducing the incidence of disease in low transmission settings, due to large reservoirs of low-level asymptomatic cases in which parasitaemia is below the limit of detection (LOD) of RDTs — roughly 100 parasites/μl. Unless the LOD of RDTs can be significantly improved, molecular methods such as LAMP or nested PCR, which demonstrate LODs as low as 0.02 parasites/μl, will be of greater utility in elimination or pre-elimination settings. [130] While RDTs will still be of financial benefit in these low-transmission populations, it will only be as a platform for confirmatory diagnosis of acute infections.

## 8. Conclusions and Future Directions

Rapid diagnostic tests show great potential for meeting the need for low-cost, rapid, point-of-care confirmatory tests for symptomatic cases of infectious disease. Although at present only malaria RDTs have achieved appreciable market penetration and technological maturity, in coming years the test format will be successfully adapted for the diagnosis of a broad variety of vector-borne tropical diseases, sexually transmitted infections and even noncommunicable conditions. The identification of a new generation of biomarkers via the sensitive “omics” analysis of complex patient samples will enable the sensitive and specific diagnosis of these illnesses at the point-of-care, reducing morbidity and mortality, and informing epidemiological models and elimination campaigns.

Throughout this process, the considerable infrastructure and regulatory development that has occurred in the roll-out of malaria RDTs will hopefully serve to jump-start research, development, and validation of rapid diagnostic tests for these new disease targets, as well as the creation of efficient, high-coverage supply chains. By identifying the myriad technical, biological, social, infrastructural, regulatory, and economic issues that have contributed to the slow market acceptance of RDTs thus far, and by proposing context-appropriate solutions, it is hoped that the format can be fundamentally improved, and that the same pitfalls can be avoided for other disease contexts.

These fundamental paradigm shifts may already be underway. In the near future, microfluidics-, paper- or thread-based formats might supplant those based on nitrocellulose membranes. [131–134] Devices based on these novel modalities can be manufactured at scale using existing infrastructure, and can be engineered to support internal, multistep sample processing, allowing for facile user operation. [135] Additionally, antibodies may soon be replaced by thermostable, bio-orthogonal non-immunoglobulin affinity agents, reducing the cost of test production and increasing test stability in infrastructure-poor contexts. [56, 136] RDTs may also be adapted for the multiplexed detection of disparate pathogens, [87, 103] allowing for the efficient diagnosis of patients with non-specific clinical presentations. Finally, the spread of RDTs may be supported by technologies leveraging the broad availability of cell phones, for improved sensitivity and reduced subjectivity in test line interpretation. [137]

Whatever the ultimate direction of development, the progressive improvement of rapid diagnostic tests will undoubtedly continue, driven by the format's potential applications in developed contexts (e.g., veterinary science, environmental monitoring, biosecurity and triage testing/in-home self-monitoring); by the rapidly expanding, government-subsidized market for low-cost diagnostics; and by the innate potential of RDTs to improve global health by enabling point-of-care diagnosis of infectious disease.

## 9. Compliance with Ethical Research Standards

The authors declare no conflicts of interest. No part of this study was performed on any human or animal subjects.
